# Optimal timeframe for achieving biochemical remission in Crohn’s disease patients treated with first-line biologics: A retrospective multicenter study

**DOI:** 10.1097/MD.0000000000040074

**Published:** 2024-10-11

**Authors:** Ji Eun Na, Yong Eun Park, Jongha Park, Tae-Oh Kim, Jong Hoon Lee, Su Bum Park, Soyoung Kim, Seung Bum Lee

**Affiliations:** a Department of Internal Medicine, Inje University Haeundae Paik Hospital, Busan, South Korea; b Department of Internal Medicine, Dong-A University College of Medicine, Busan, South Korea; c Department of Internal Medicine, Pusan National University School of Medicine and Research Institute for Convergence of Biomedical Science and Technology, Pusan National University Yangsan Hospital, Yangsan, South Korea; d Department of Gastroenterology, Ulsan University Hospital, University of Ulsan College of Medicine, Ulsan, South Korea.

**Keywords:** adequate timeframe, C-reactive protein, first-line biological therapy, moderate to severe Crohn’s disease

## Abstract

Predicting treatment response in Crohn’s disease (CD) patients initiating biological therapy is crucial. The first step involves considering symptom control and normalization of C-reactive protein (CRP). However, data on the actual rates of achieving CRP normalization and the appropriate timeframe are lacking. Therefore, we aim to investigate the rate of attaining CRP normalization and identify its optimal timeframe in CD patients initiating biological therapy. In this retrospective multi-center study, we analyzed moderate to severe CD patients initiating biological therapy from January 2012 to July 2023. The primary outcome was the rate and timeframe for achieving CRP normalization. Secondary outcomes included clinical outcomes in patients who achieved CRP normalization and factors associated with early CRP normalization. Of 183 patients, 123 (67.2%) achieved CRP normalization, with a median duration of 3.8 months (interquartile range 1.4 to 7.4 months). The duration and value difference for CRP normalization between anti-tumor necrosis factor agents, ustekinumab, and vedolizumab were statistically insignificant. Cumulative rates of CD-related hospitalization, intestinal resection, and drug discontinuation over 8 years were 11.4%, 2.4%, and 12.2%, respectively. The duration of CRP normalization correlates with drug discontinuation (area under the curve: 0.64). Treatment with 5-aminosalicylic acid (HR 2.77; 95% confidence interval [CI] 1.26–6.11) and high albumin level (HR 1.64, 95% CI 1.04–2.61) favored early CRP normalization, whereas structuring behavior less likely than inflammatory behavior (HR 0.43, 95% CI 0.19–0.96). We have provided the actual rate of achieving CRP normalization and its appropriate timeframe as an initial target in CD treatment.

## 1. Introduction

Crohn’s disease (CD), one of the inflammatory bowel diseases, is characterized by chronic relapses and remissions, and patients may experience complications such as stenosis, fistulas, and abscesses and require subsequent abdominal surgery.^[1]^ In moderate to severe CD patients receiving biologic therapy, between 7% and 25% of patients undergo abdominal surgery within the third year of treatment,^[2–4]^ and approximately 25% to 27% of patients experience discontinuation of biologic therapy.^[5,6]^ Because these events significantly impact patients’ quality of life, managing disease activity and predicting relapses is crucial. To enhance these prognoses, the Selecting Therapeutic Targets in Inflammatory Bowel Disease-II has proposed short-term, intermediate-term, and long-term goals, including symptom control and normalization of C-reactive protein (CRP) levels, reduction in fecal calprotectin (a marker reflecting the degree of intestinal inflammation), and endoscopic healing, respectively.^[7]^

For CD patients initiating biologic therapy for the first time, achieving short-term goals as the first step, including symptom control and CRP normalization, plays a pivotal role in predicting treatment response for patients and clinicians. Additionally, it is known to be associated with attaining long-term goals with endoscopic healing.^[8,9]^ However, it is not clear how long “short term” signifies.

We need to know the appropriate timeframe and rates for achieving biochemical remission, referring to CRP normalization in this study, in CD patients initiating biologic therapy. Therefore, we planned a retrospective multi-center study to investigate the suitable timeframe and rates of achieving CRP normalization for moderate to severe CD patients starting biologic therapy. Additionally, we identified the long-term prognosis of CD patients who achieved biochemical remission.

## 2. Methods

### 2.1. Patients

This retrospective cohort study was conducted at three referral centers. It included adult patients aged 18 and above with moderate-to-severe CD who initiated biologic therapy at each hospital from January 2012 to July 2023. The following cases were excluded: (1) irregular administration of biologics due to insurance issues or noncompliance, (2) patients with CRP levels below 0.5 mg/dL before starting their first biological therapy, (3) patients who failed to maintain CRP normalization due to other diseases or infections, (4) cases where CRP normalization was confirmed after dose intensification, (5) patients with <3 months of follow-up. The Institutional Review Board of Haeundae Paik Hospital approved our protocol (File number. 2023-04-013-005). Patients’ informed consent requirement was waived as only de-identified data were collected.

### 2.2. Outcomes and assessment

The primary outcome of this study is to investigate the rate of achieving CRP normalization after initiating first-line biologics. This involves determining the duration and value difference required to attain CRP normalization. The types of biologics included in the study are antitumor necrosis factor agents (anti-TNF agents) (infliximab and adalimumab), ustekinumab, and vedolizumab. Achieving CRP normalization means maintaining CRP levels below 0.5 mg/dL for at least 6 months without using steroids. CRP normalization duration is measured from the initiation of biological therapy to the first day of achieving CRP normalization. To determine the prebiologic therapy value, we considered the worst value of CRP within the 3 months preceding the start of biologic treatment. The value on the first day of achieving CRP normalization was also recorded, and the difference between these values was used to assess the value difference.

The secondary outcomes involve confirming the clinical outcomes of patients who achieved CRP normalization and identifying factors associated with early CRP normalization. Clinical outcomes were assessed based on CD-related hospitalization, intestinal resection, and drug discontinuation after the first day of CRP normalization.

The index date was the start date of first-line biological therapy. The induction and maintenance therapy schedules and response assessment after induction therapy were conducted as follows: (1) infiniximab was administered intravenously at a dose of 5 mg/kg at weeks 0, 2, and 6, followed by administration every 8 weeks. Response assessment after induction was performed before the second dose administration; (2) adalimumab was administered subcutaneously at 160 mg at week 0, 80 mg at week 2, and 40 mg every 2 weeks. Response assessment after induction was conducted before the 3rd dose administration; (3) ustekinumab was administered intravenously at week 0, with the dose depending on body weight (260 mg in ≤55 kg, 390 mg from >55 kg to ≤85 kg, or 520 mg in >85 kg), followed by subcutaneous ustekinumab 90 mg at week 8, and maintained every 12 weeks. Response assessment after induction was performed before the 3rd dose administration; and (4) vedolizumab was administered intravenously at a dose of 300 mg at weeks 0, 2, and 6, followed by administration every 8 weeks. Response assessment after induction was conducted after the 3rd dose administration. In South Korea, biological therapies for moderate to severe CD patients follow the above schedules based on insurance policies. Maintenance therapy is approved based on a clinical response before and after induction using the Crohn’s Disease Activity Index (CDAI). Patients included in the study were followed up continuously until the last administration date of first-line biologics or the last outpatient visit date.

### 2.3. Covariates

Information on the following variables was collected retrospectively from medical records: gender, age at diagnosis, age at the initiation of the first biological therapy, disease duration, location, behavior, perianal disease, history of intestinal resection, CDAI before first biological therapy, concomitant medicines [5-aminosalicylic acid, steroid, and immunomodulators], laboratory data (CRP, hemoglobin, and albumin), and follow-up duration.

### 2.4. Statistical analysis

Continuous variables were presented as mean ± standard deviation and compared using a one-way analysis of variance or the Kruskal–Wallis test. Categorical variables were expressed as n (%) and compared using the chi-squared or Fisher exact test. When comparing the rates of achieving CRP normalization between anti-TNF agents, ustekinumab, and vedolizumab, we applied Bonferroni correction to account for multiple comparisons and considered a significance of *P* < .0167. The duration (in months) and difference of value (the difference between the prebiologic therapy CRP value and the CRP value on the first day of confirmed CRP normalization) among anti-TNF agents, ustekinumab, and vedolizumab were assessed using the Kruskal–Wallis test. To investigate the association between the timeframe to CRP normalization and drug discontinuation in CD patients who achieved CRP normalization, we used the receiver operating characteristic curve. To find the optimal cutoff, we assess the area under the curve (AUC) and calculate sensitivity, specificity, positive predictive value (PPV), and negative predictive value (NPV) at the optimal cutoff. Cox proportional hazard analysis was used to identify factors associated with achieving early CRP normalization. We included only those factors with a univariate analysis *P* value < .05 in the multivariate analysis. The cumulative rates of clinical outcomes (CD-related hospitalization, intestinal resection, and drug discontinuation) in patients who achieved CRP normalization were assessed at 2 years, 4 years, 6 years, and 8 years using Kaplan–Meier survival analysis. A *P* value < .05 was considered statistically significant. Statistical analysis was performed using SPSS Statistics 25.0 and *R*-4.2.2.

## 3. Result

### 3.1. Baseline characteristics

Out of the 223 adult patients with moderate to severe CD who initiated their first biological therapy, a total of 183 patients were included in the study after excluding 40 patients who met the previously mentioned exclusion criteria. Among these patients, 135 (73.8%) received anti-TNF agents, 42 (23.0%) received ustekinumab, and 6 (3.3%) received vedolizumab (Table 1). In baseline characteristics, there was a higher proportion of males (72.7%) than females (27.3%). The mean age at the start of the first biological therapy was 28 years, with the ustekinumab group having an older mean age of 31 years compared to 26 years for the anti-TNF agents group and 23 years for the vedolizumab group. No significant differences were observed between the anti-TNF agents, ustekinumab, and vedolizumab groups regarding disease duration, location, behavior, perianal disease, or a history of intestinal resection. The CDAI before starting the first-line biologics was 265.5, with the ustekinumab group showing a significantly higher value than the group receiving anti-TNF agents. Within 3 months before beginning the first-line biologics, more than half of the patients (55.7%) had concomitant steroid use, and most patients (85.2%) were concurrently taking immunomodulators. The mean CRP before starting biologic therapy was 2.2 mg/dL, with no significant differences among the anti-TNF agents, ustekinumab, and vedolizumab groups (2.4 mg/dL, 1.7 mg/dL, and 2.5 mg/dL, respectively). The follow-up duration was significantly longer in the anti-TNF agents group (mean 3.5 years) compared to ustekinumab (mean 2.2 years) and vedolizumab (mean 0.6 years) groups.

**Table 1:** Baseline characteristics of the patients at the initiation of biological therapy.

	Overall population (N = 183)	Anti-TNF agents (N = 135, 73.8%)	Ustekinumab (N = 42, 23.0%)	Vedolizumab (N = 6, 3.3%)	*P* values*^,^ **^,^ ***
Male/female	133/50 (72.7/27.3)	98/37 (72.6/27.4)	31/11 (73.8/26.2)	4/2 (66.7/33.3)	<.001* <.001** .013***
Age at diagnosis†	23 (12)	22 (11)	28 (15)	22 (5)	.009* .591** .043***
Age at first biological therapy†	28 (14)	26 (13)	31 (16)	23 (15)	.007* .533** .098***
Disease duration, years†	2.7 (6.9)	2.7 (6.7)	2.8 (8.8)	2.7 (12.8)	.681¹
Disease location Colon Ileum Ileocolon	16 (8.7) 64 (35.0) 103 (56.3)	13 (9.6) 43 (31.9) 79 (58.5)	2 (4.8) 18 (42.9) 22 (52.4)	1 (16.7) 3 (50.0) 2 (33.3)	.372^4^
Disease behavior Inflammatory Stricturing Penetrating	116 (63.4) 41 (22.4) 26 (14.2)	86 (63.7) 27 (20.0) 22 (16.3)	28 (66.7) 11 (26.2) 3 (7.1)	2 (33.3) 3 (50.0) 1 (16.7)	.188^4^
Perianal disease	76 (41.5)	61 (45.2)	13 (31.0)	2 (33.3)	.216^4^
History of intestinal resection	34 (18.6)	26 (19.3)	6 (14.3)	2 (33.3)	.407^4^
CDAI before first biological therapy† (missing values in 5 patients)	265.5 (66.8) (N = 178)	262.0 (59.0) (N = 131)	278.0 (83.5) (N = 41)	283.5 (88.5) (N = 6)	.019* .313** .987***
Concomitant therapies Steroid Immunomodulators 5-ASA	102 (55.7) 156 (85.2) 139 (76.0)	72 (53.3) 115 (85.2) 105 (77.8)	27 (64.3) 36 (85.7) 30 (71.4)	3 (50.0) 5 (83.3) 4 (66.7)	.464^4^ .987^3^ .570^4^
Laboratory data† CRP Hemoglobin Albumin	2.2 (2.7) 12.5 (2.6) 3.8 (0.7)	2.4 (3.3) 12.5 (2.4) 3.8 (0.7)	1.7 (1.9) 13.2 (2.5) 4.0 (0.7)	2.5 (4.1) 10.6 (2.2) 3.3 (0.6)	.094¹ .117* .055** .014*** .009* .049** .007***
Follow-up duration, years†	2.9 (3.1)	3.5 (3.6)	2.2 (1.5)	0.6 (0.9)	<.001* <.001** .001***

Values are expressed as n (%) unless otherwise specified.

5-ASA = 5-aminosalicylic acid, anti-TNF = antitumor necrosis factor, CDAI = Crohn’s Disease Activity Index, CRP = C-reactive protein.

† Mean (standard deviation) presented for continuous variables.

* *P* values were a comparison between anti-TNF agents and ustekinumab.

** *P* values were a comparison between anti-TNF agents and vedolizumab.

*** *P* values were a comparison between ustekinumab and vedolizumab.

### 3.2. The timeframe of C-reactive protein normalization

**Figure 1.**
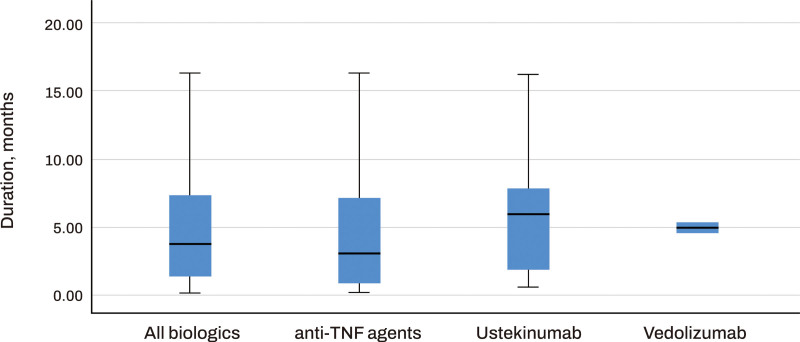
The timeframe for achieving C-reactive protein normalization of biologics.

Out of the 183 patients, CRP normalization was confirmed in 123 patients (67.2%). There was a significant difference in the rate of achieving CRP normalization among the 3 groups, with 65.9% in the anti-TNF agents group, 76.2% in the ustekinumab group, and 33.3% in the vedolizumab group. The highest rate was observed in the ustekinumab group (Table 2). The median time to achieve CRP normalization for all patients was 3.8 months, and the value difference was 1.8 mg/dL. There were no significant differences in the duration for completing CRP normalization among the 3 groups, with a median of 3.1 months for the anti-TNF agents group, 6.1 months for the ustekinumab group, and 5.1 months for the vedolizumab group (Fig. 1). The difference in value showed no significant differences among the groups, with difference values of 2.1 mg/dL, 1.5 mg/dL, and 2.5 mg/dL, respectively.

**Table 2:** C-reactive protein normalization rates after initiation of biological therapy corresponding duration and difference of values in patients with achieving C-reactive protein normalization.

	Overall patients (N = 183)	Anti-TNF agents (N = 135)	Ustekinumab (N = 42)	Vedolizumab (N = 6)	*P* *	*P* **	*P* ***
CRP normalization number (%)	123 (67.2)	89 (65.9)	32 (76.2)	2 (33.3)	<.001	<.001	<.001
Duration, months†	3.8 (1.4, 7.4)	3.1 (1.0, 7.2)	6.1 (2.0, 7.9)	5.1 (4.7, 5.4)	.064		
Difference of value, mg/dL†	1.8 (1.1, 3.7)	2.1 (1.1, 3.9)	1.5 (0.9, 2.2)	2.5 (2.3, 2.7)	.167		

Anti-TNF = antitumor necrosis factor; CRP = C-reactive protein.

† Median (interquartile range) presented.

* *P* values were a comparison between anti-TNF agents and ustekinumab.

** *P* values were a comparison between anti-TNF agents and vedolizumab.

*** *P* values were a comparison between ustekinumab and vedolizumab.

### 3.3. Clinical outcomes and associating factors

**Figure 2.**
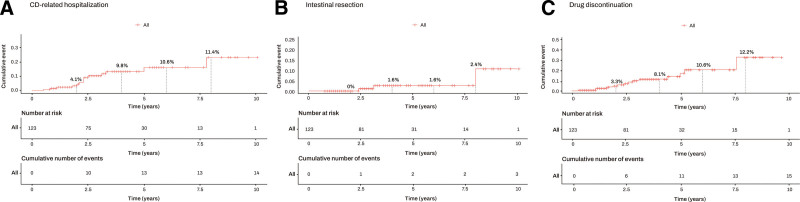
Clinical outcomes in patients with achieving C-reactive protein normalization. (A) Hospitalization, (B) intestinal resection, and (C) biologics discontinuation.

Out of the 183 patients in the study, clinical remission after induction biological therapy was confirmed in 136 patients, of which 99 (72.8%) showed CRP normalization. Among the 47 patients who did not achieve clinical remission after induction biological therapy, 24 (51.1%) showed CRP normalization (*P* = .006). Of the 123 patients who achieved CRP normalization, clinical outcomes, including CD-related hospitalization, intestinal resection, and drug discontinuation, were assessed. During the follow-up period, CD-related hospitalization occurred in 14 patients (11.4%), intestinal resection in 3 patients (2.4%), and drug discontinuation in 15 patients (12.2%). Biologics discontinuation among these 15 patients was primarily due to secondary loss of response in 14 cases (Table 3). The cumulative occurrence rates for CD-related hospitalization were 4.1% at 2 years, 9.8% at 4 years, 10.6% at 6 years, and 11.4% at 8 years. Intestinal resection had cumulative occurrence rates of 0% at 2 years, 1.6% at 4 years, 1.6% at 6 years, and 2.4% at 8 years. Drug discontinuation had cumulative occurrence rates of 3.3% at 2 years, 8.1% at 4 years, 10.6% at 6 years, and 12.2% at 8 years (Fig. 2).

**Table 3:** Clinical outcomes in patients with achieving C-reactive protein normalization.

	Overall patients (N = 123)	Anti-TNF agents (N = 89)	Ustekinumab (N = 32)	Vedolizumab (N = 2)
Hospitalization	14 (11.4)	11 (12.4)	3 (9.4)	0 (0.0)
Intestinal resection	3 (2.4)	3 (3.4)	0 (0.0)	0 (0.0)
Biologics discontinuation	15 (12.2)	14 (15.7)	0 (0.0)	1 (50.0)
Secondary loss of response	14 (11.4)	13 (14.6)	0 (0.0)	1 (50.0)
Intolerance	1 (0.0)	1 (0.0)	0 (0.0)	0 (0.0)

Values are expressed as n (%).

**Figure 3.**
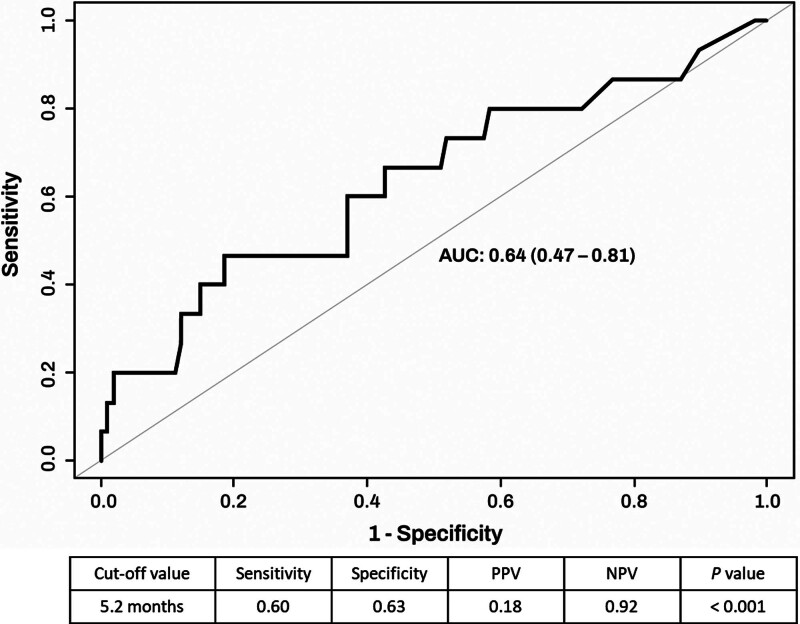
Association between C-reactive protein normalization duration and biologics discontinuation.

When analyzing the association between the duration of achieving CRP normalization and biological therapy discontinuation among the 123 patients (67.2%) who achieved CRP normalization, the AUC was 0.64, indicating acceptable performance (Fig. 3). The optimal cutoff for the timeframe of CRP normalization predicting biologics discontinuation was found to be 5.2 months. This optimal cutoff of 5.2 months had a sensitivity of 0.60, specificity of 0.63, PPV of 0.18, and NPV of 0.92, demonstrating high NPV.

Early CRP normalization was defined as achieving CRP normalization within 3.8 months, representing the median duration among patients who achieved CRP normalization. Factors favoring the achievement of early CRP normalization included treatment with 5-aminosalicylic acid [hazard ratio (HR) 2.77; 95% confidence interval (CI) 1.26–6.11] and higher albumin levels (HR 1.64, 95% CI 1.04–2.61) (Table 4). Conversely, patients exhibiting stricturing behavior were less likely to achieve early CRP normalization than those with inflammatory behavior (HR 0.43, 95% CI 0.19–0.96).

**Table 4:** Associating factors for achieving early C-reactive protein normalization.

	Univariate	Multivariate		
	*P*	HR	95% CI	*P*
Biologics Anti-TNF agents* Ustekinumab Vedolizumab	.168 .964			
Male*/female	.047	1.63	0.96–2.77	.073
Age at diagnosis	.470			
Age at first biological therapy	.483			
Disease duration, years	.950			
Disease location Colon* Ileum Ileocolon	.472 .862			
Disease behavior Inflammatory* Stricturing Penetrating	.035 .438	0.43 1.20	0.19–0.96 0.61–2.35	.040 .601
Perianal disease no*/yes	.052			
History of intestinal resection no*/yes	.700			
CDAI before first biological therapy	.351			
Steroid no*/yes	.790			
Immunomudoulators no*/yes	.330			
5-ASA no*/yes	.011	2.77	1.26–6.11	.011
CRP	.440			
Hemoglobin	.727			
Albumin	.016	1.64	1.04–2.61	.035

5-ASA = 5-aminosalicylic acid, anti-TNF = antitumor necrosis factor, CDAI = Crohn’s Disease Activity Index, CI = confidence interval, CRP = C-reactive protein, HR = hazard ratios.

* Reference.

## 4. Discussion

We found that the rate of achieving CRP normalization was 67.2%, with a median duration of 3.8 months. While the rate of CRP normalization was higher in the ustekinumab group compared to the anti-TNF agents and vedolizumab groups, it is important to note that several factors, including underlying disease activity, may influence the CRP normalization rates of each biological therapy. Furthermore, the type of biologics was not a significant factor associated with achieving CRP normalization in multivariable analysis. The duration for completing CRP normalization and the difference in values did not show significant differences between the biologics. However, it is worth mentioning that the absolute timeframe for achieving CRP normalization appeared shorter for the anti-TNF agents group than for the ustekinumab and vedolizumab groups. When assessing whether the duration of CRP normalization in patients who achieved it could predict drug discontinuation, an AUC of 0.64 was considered acceptable,^[10]^ and the optimal timeframe was found to be 5.2 months. Within this timeframe, it was possible to predict drug discontinuation with a sensitivity of 0.60, specificity of 0.63, PPV of 0.18, and NPV of 0.92, indicating relatively high NPV. Among the 123 patients who achieved CRP normalization, 11.4% experienced CD-related hospitalization, 2.4% underwent intestinal resection, and 12.2% experienced biological therapy discontinuation during the follow-up period. This highlights the need for additional timeframes for intermediate- and long-term targets, even after achieving short-term targets.

This paper represents the first study to investigate the rate of CRP normalization as one of the short-term targets following the initiation of biologic therapy in moderate to severe CD patients and to identify the optimal timeframe for predicting drug discontinuation. While rates of maintaining clinical remission as other short-term targets^[7]^ 1 or 2 years after the initiation of biologics have been reported to be <50%,^[11–14]^ it is essential to note that among patients who maintained clinical remission, the rate of preserving CRP normalization is approximately 44%.^[15]^ This phenomenon, often called silent CD, is characterized by the absence of clinical symptoms despite ongoing inflammation and is associated with poor clinical outcomes.^[16,17]^ Therefore, this paper holds clinical significance as it aims not only to identify the attainment of CRP normalization as the primary goal but also to determine an appropriate timing associated with prognosis.

Biochemical remission has been reported to have a favorable correlation with mucosal healing (MH) and transmural healing (TH).^[8]^ Indeed, the treatment goals in CD have been evolving to TH over MH.^[18]^ However, in meta-analyses based on randomized controlled trials, the rate of achieving MH did not exceed 30%,^[19]^ and the rate of TH varies widely depending on the evaluation tool and timing, ranging from 14% to 42.4%.^[20,21]^ In practice, the actual attainment rates of each goal have been low, and research studies have employed varying methodologies. Furthermore, unlike ideal scenarios, changing biologics does not always yield better results in practice. Insurance policies in different countries can also limit the switching and reversion of biological therapies. To address these challenges, our study aimed to examine the rate of achieving the initial treatment goal, which is CRP normalization, and to identify the optimal timing for this achievement. We also determined that the appropriate timeframe for predicting drug discontinuation, based on the duration of achieving CRP normalization, is 5.2 months.

Among the 123 patients who achieved CRP normalization, the cumulative incidence rates of CD-related hospitalization, intestinal resection, and drug discontinuation at an 8-year follow-up were 11.4%, 2.4%, and 12.2%, respectively. It has been reported that in patients initiating biologics, the rate of hospitalization at 6 years is 38.7%, and the rate of intestinal surgery is 12.2%.^[2]^ Drug discontinuation rates within 3 years of treatment initiation have been reported to range from 13.3% to 32.8%.^[22–24]^ In a cohort where only patients with confirmed CRP normalization were tracked for a median of 8 years, hospitalization and intestinal resection rates were 38.9% and 13.3%, respectively.^[15]^ Still, this cohort differed significantly, with only approximately 7% of patients receiving biological therapies from our study. Compared to previous reports,^[4]^ the clinical outcomes of patients with confirmed CRP normalization in this paper demonstrated a more favorable prognosis. At the same time, it underscores the appropriate timeframes and acceptable values for subsequent treatment goals are necessary after achieving short-term targets.

This study has several limitations. First, being a retrospective study, the timing of response assessments after induction and the tracking intervals during maintenance therapy varied among the different biologics. In most cases, patients are generally monitored with intervals of approximately 2 to 3 months for assessing disease activity, including CRP values. Second, we lack a comparison between different biologics. However, our primary focus was not on comparing the efficacy of different biologics but rather on providing data regarding the rate of achieving CRP normalization and identifying its appropriate timeframe in CD patients starting biologic therapy for the first time. Third, this study only targeted bio-naïve patients, which may limit its generalizability. Fourth, due to the small number of clinical events, we were only able to analyze an optimal timeframe related to drug discontinuation.

In conclusion, CRP normalization was achieved in approximately two-thirds of the CD patients initiating the first biological therapy. The optimal timeframe associated with drug continuation was within 8 months in patients who achieved CRP normalization. Clinical outcomes of patients who achieved CRP normalization were relatively favorable. Based on our findings, future research will be needed to establish appropriate timeframes and standardized values for short-term, intermediate-term, and long-term treatment targets.

## Author contributions

**Conceptualization:** Tae-Oh Kim, Ji Eun Na.

**Data curation:** Ji Eun Na, Su Bum Park, Soyoung Kim, Seung Bum Lee.

**Formal analysis:** Ji Eun Na.

**Funding acquisition:** Tae-Oh Kim.

**Methodology:** Ji Eun Na.

**Resources:** Jongha Park, Tae-Oh Kim, Su Bum Park, Seung Bum Lee.

**Supervision:** Yong Eun Park, Jongha Park, Tae-Oh Kim, Jong Hoon Lee, Su Bum Park, Soyoung Kim, Seung Bum Lee.

**Visualization:** Ji Eun Na.

**Writing – original draft:** Ji Eun Na.

**Writing – review & editing:** Ji Eun Na, Tae-Oh Kim.
